# Extra-gastrointestinal stromal tumor arising in the lesser omentum with a platelet-derived growth factor receptor alpha (PDGFRA) mutation: a case report and literature review

**DOI:** 10.1186/s12957-020-01961-1

**Published:** 2020-07-23

**Authors:** Kohei Kanamori, Yukinori Yamagata, Yoshitaka Honma, Keiichi Date, Takeyuki Wada, Tsutomu Hayashi, Sho Otsuki, Shigeki Sekine, Takaki Yoshikawa, Hitoshi Katai, Toshiro Nishida

**Affiliations:** 1 Gastric Surgery Division, National Cancer Center Hospital, 5-1-1 Tsukiji, Chuo-ku, Tokyo, 104-0045 Japan; 2 Head and Neck Medical Oncology Division and Gastrointestinal Medical Oncology Division, National Cancer Center Hospital, 5-1-1 Tsukiji, Chuo-ku, Tokyo, 104-0045 Japan; 3 Division of Pathology and Clinical Laboratories, National Cancer Center Hospital, 5-1-1 Tsukiji, Chuo-ku, Tokyo, 104-0045 Japan

**Keywords:** Extra-gastrointestinal stromal tumor, Omentum, Platelet-derived growth factor receptor alpha

## Abstract

**Background:**

Gastrointestinal stromal tumors (GIST) arising from sites other than the gastrointestinal (GI) tract, termed extra-gastrointestinal stromal tumors (EGIST), are rare. Among EGIST, those with platelet-derived growth factor receptor alpha (*PDGFRA*) mutations are even rarer, with only a few cases reported. About 80% of GIST has *KIT* mutations, and 10% of GIST have *PDGFRA* mutations, which commonly affect the TK2 domain (exon 18). Among the exon 18 mutations, the *D842V* substitution is limited to gastric GIST. In EGIST, the degree of *KIT* and *PDGFRA* mutations varies on where the location of the tumor is, and it is suggested that omental EGIST is similar to gastric GIST. Adjuvant imatinib therapy is recommended for high-risk GIST; however, it is known that imatinib is less effective against GIST with a *PDGFRA D842V* mutation.

**Case presentation:**

A 75-year-old man was referred to our hospital with an extrinsic tumor of the lesser curvature of the gastric body. Intraoperative findings showed a tumor located outside of the lesser omentum with no connection between the tumor and the gastric wall. The tumor was subsequently resected. Pathological examination indicated a GIST arising in the lesser omentum measuring 70 mm in its longer dimension. Because the tumor had a PDGFRA mutation (D842V substitution), imatinib was suspected to lack efficacy to the tumor. Thus, although the tumor was considered clinically to have a high risk of recurrence, adjuvant imatinib therapy was not indicated. The patient has been free of recurrence for 29 months since the surgery.

**Conclusion:**

We described a case of EGIST with a PDGFRA mutation arising in the lesser omentum. And we reviewed 57 cases of omental EGIST and showed that the clinicopathological characteristics and mutation status in omental EGIST were very similar to gastric GIST. In particular, *PDGFAR D842V* mutation rate in omental EGIST seemed as high as that in gastric GIST. These results suggested that omental EGIST is strongly related to gastric GIST, so the behavior of omental EGIST might be akin to gastric GIST. However, further studies are required to determine the prognosis and the necessity of adjuvant therapy for EGIST with a *PDGFRA* mutation.

## Background

A gastrointestinal stromal tumor (GIST) is a relatively rare mesenchymal tumor of the gastrointestinal (GI) tract and is defined as a spindle cell or epithelioid neoplasm expressing a c-kit (CD117) immunophenotype, which is the tyrosine kinase (TK) component [1]. GIST usually has *KIT* mutations; however, a few have been reported with mutations in platelet-derived growth factor receptor alpha (*PDGFRA*) [2]. As mentioned above, GIST usually arises from the GI tract; however, entity GIST termed extra-gastrointestinal stromal tumors (EGIST) may arise in sites other than the GI tract. EGIST represent less than 5% of all stromal tumors [3]. Similar to GIST arising in the GI tract, EGIST usually show *KIT* mutations; however, EGIST with *PDGFRA* mutations appear to be rare. We report a case of EGIST with a *PDGFRA* mutation arising in the lesser omentum.

## Case presentation

**Fig. 1:**
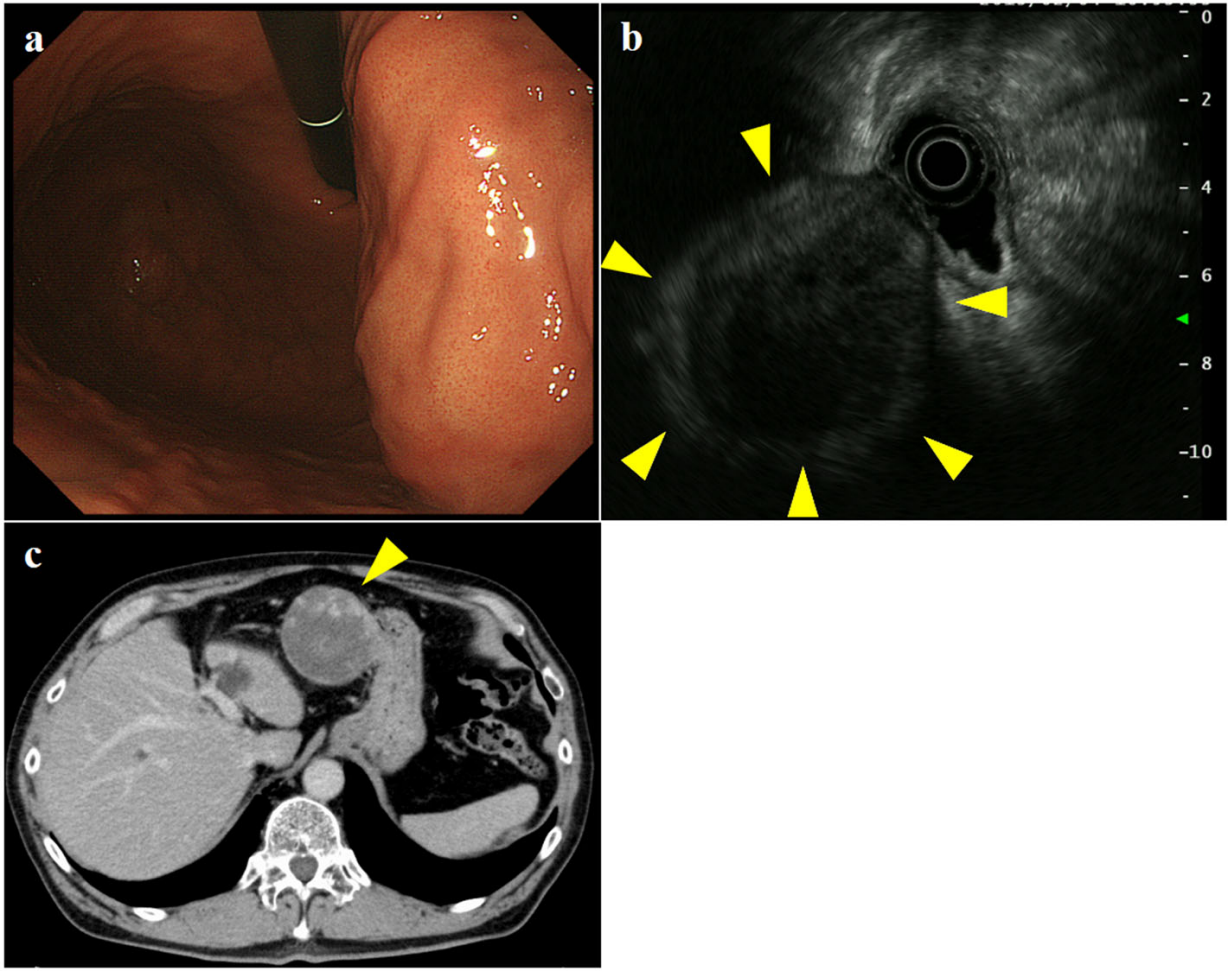
Findings of preoperative examination. Esophagogastroduodenoscopy (EGD) revealed a gently sloping protruding lesion with smooth surface mucosa, located anterior to the lesser curvature of the gastric body. Ultrasonic endoscopy (EUS) showed an extra-stomach hypoechoic tumor with a diameter of 79 × 58 mm that seemed to be connected to the smooth muscle layer of the stomach. Enhanced computed tomography (CT) revealed a solid tumor, 70-mm in diameter, which also seemed to be connected to the lesser curvature side of the gastric body

**Fig. 2:**
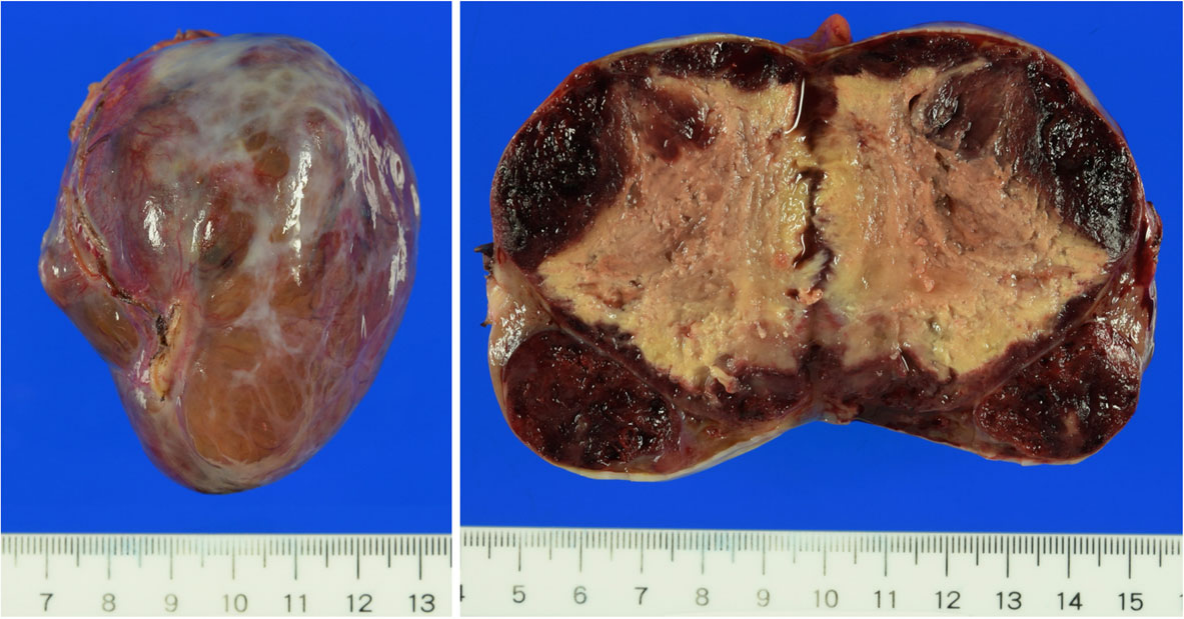
Macroscopic tumor findings. The tumor was very close to the stomach; however, the pseudocapsule of the tumor was fully kept without rupture and the resected specimen did not contain any gastric component. The tumor had a smooth surface and consisted of a solid component with extensive hemorrhage and degeneration

**Fig. 3:**
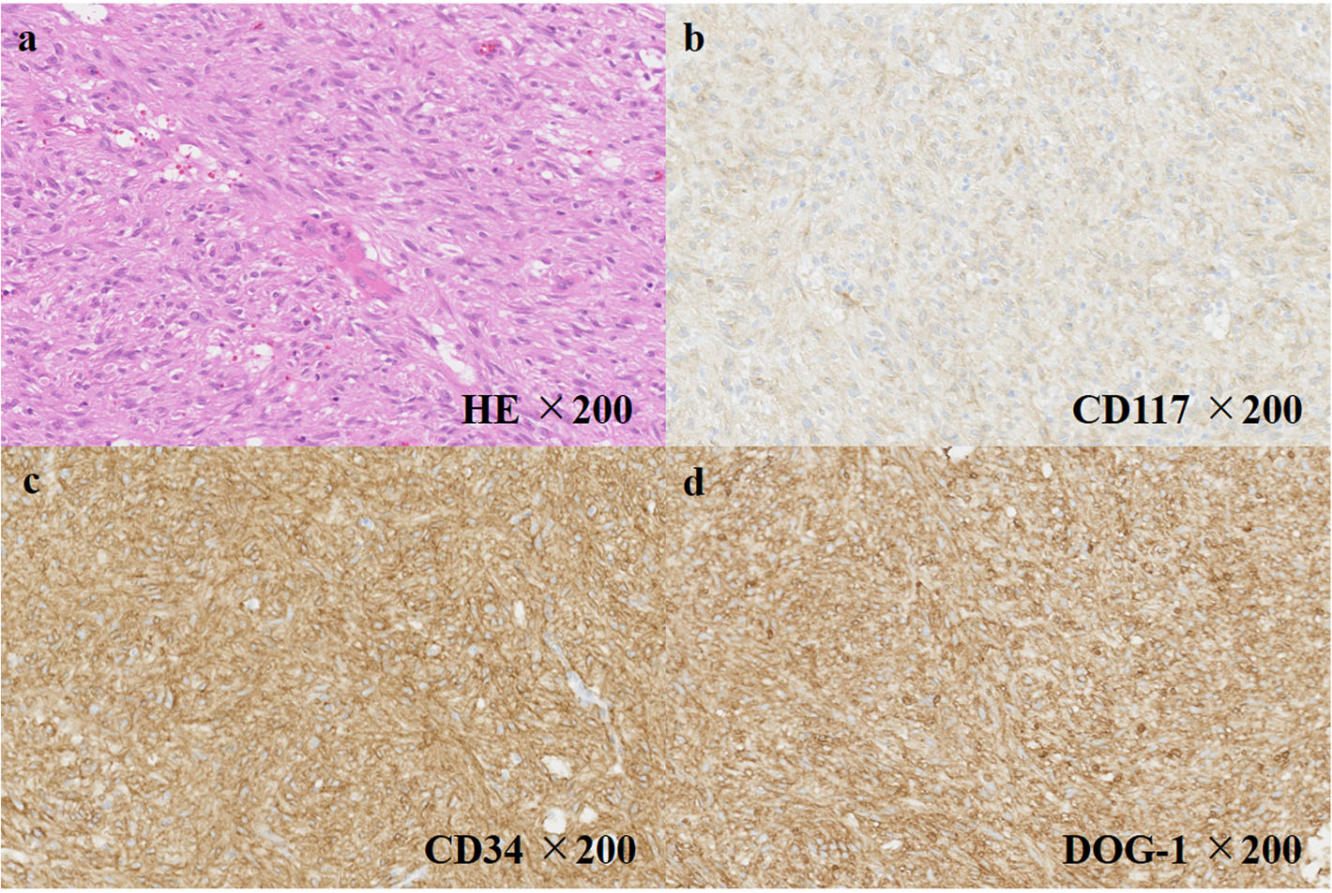
Histological and immunohistochemical tumor findings. Hematoxylin and eosin (HE) staining showing spindle-shaped tumor cells (**a**). Positive immunohistochemistry for KIT (**b**), CD34 (**c**), and DOG-1 (**d**)

A 75-year-old man was referred to our hospital due to an abdominal mass detected by abdominal ultrasonography performed during a health check. He had histories of diabetes mellitus, hypertension, hyperlipidemia, and gallstones. Esophagogastroduodenoscopy (EGD) revealed a gently sloping protruding lesion with smooth surface mucosa, located anterior to the lesser curvature of the gastric body (Fig. 1a). Endoscopic ultrasonography (EUS) showed a submucosal hypoechoic tumor with a diameter of 79 × 58 mm that seemed to be connected to the smooth muscle layer of the stomach (Fig. 1b). Due to the risk of dissemination, fine-needle aspiration biopsy was not performed. Enhanced computed tomography (CT) revealed a solid tumor, 70-mm in diameter, which also seemed to be connected to the lesser curvature side of the gastric body (Fig. 1c). Based on the results of EGD, EUS, and CT, we diagnosed this tumor as an extramural growth-type gastric submucosal tumor highly suspicious of GIST and planned surgical resection. Resection of the tumor through laparotomy was performed. The tumor was located at lesser omentum, measured 70 mm in its longer dimension, and was connected to the lesser omentum. The tumor was very close to the stomach; however, no connection was present between the tumor and gastric wall, and it could be resected without rupturing the pseudocapsule and did not contain any gastric component. The tumor had a smooth surface; the cut surface was solid with extensive hemorrhage and degeneration (Fig. 2). Histological examination showed the proliferation of epithelioid to spindle-shaped tumor cells with oval, rather uniform nuclei (Fig. 3a). The mitotic count was less than 1 per 50 high-power fields (HPFs). The tumor cells were immunohistochemically positive for KIT, CD34, and discovered on GIST-1 (DOG-1) but negative for S-100 and alpha-SMA (Fig. 3b–d). The Ki-67 labeling index was 3.3%. We finally diagnosed the tumor as an EGIST originating from the lesser omentum.

According to Joensuu’s classification, the size was 70 mm (over 50 mm) and the location of the tumor was “nongastric,” we considered this tumor high-risk [4]. Then, to determine the indication of adjuvant therapy, we performed a genetic examination of the tumor, which revealed no *KIT* mutation but instead revealed a *PDGFRA D842V* substitution. Based on a previous report that imatinib was less effective against GIST with a *PDGFRA D842V* mutation [2], adjuvant imatinib therapy was not indicated for the present patient. Instead, the patient has been carefully followed up by CT examination every 6 months and remains free of recurrence for 29 months after surgery.

## Discussion

GISTs are specific mesenchymal tumors that are considered to originate from the interstitial cells of Cajal (ICCs), the pacemaker of the peristaltic movement of the GI tract [5]. Hence, they usually occur throughout the length of the GI tract, from the esophagus to the anus. However, there are GISTs arising in sites other than the GI tract, such as the omentum, mesentery, and retroperitoneum. Those GISTs are called EGIST [3].

GIST is a relatively rare tumor, which occurs in 6.8–14.5 cases per million people per year [6]. The common primary sites for GIST are the stomach (60–70%), jejunum (25–30%), colorectum (5–15%), and duodenum (5%) [7, 8]. EGISTs are estimated less than 10% of all GISTs, and they occur in the mesentery (7.7–29.4%), retroperitoneum (25.5–25.6%), and omentum (7.7–15.7%) [3, 9, 10]. Hence, it is suggested that this case, EGIST in the omentum, seems to be extremely rare.

Some studies have reported that there are similar epidemiological and histological findings between GIST and EGIST [9, 11]. We searched all articles on EGIST in the PubMed database, published up to December 2019 using the following keywords: extra-gastrointestinal stromal tumor, EGIST, gastrointestinal stromal tumor, GIST, omental, and omentum. We also examined the reference sections of published articles. We excluded articles with obvious irrelevance and finally acquired and reviewed 57 cases of omental EGIST from 25 series of small case series and case reports. The characteristics of these cases were summarized and compared with whole GIST in Table 1 [6, 10, 12–35]. The age at onset, gender ratio, and histological form of omental EGIST were very similar to those of GIST.

**Table 1:** Clinicopathological findings of patients with omental extra-gastrointestinal stromal tumors (*n* = 57) [6, 10, 12–35]

	*Omental EGIST* (*n = 57*)	*Whole GIST*
**Age (years, median, range)**	63 (27–89)	60s
**Sex (male/female)**	32/25 (1.28:1)	1:1
**Tumor size (cm, median, range)**	15 (1–36)	―
**Histologic morphology**		
Spindle	30 (53.6%)	70%
Epithelioid	16 (28.6%)	20%
Mixed	8 (14.3%)	10%
Myxoid	2 (3.6%)	
**Mitotic rate (per 50 HPFs, median, range)**	3 (0–115)	―

*KIT* and/or *PDGFRA* mutations are thought to be the main drivers of GIST [2]. About 80% of GISTs have *KIT* mutations, of which the most common are in-frame deletions or insertions or missense mutations within the juxtamembrane domain (exon 11). And 10% of GIST have *PDGFRA* mutations, which commonly affect the TK2 domain (exon 18) [2, 36–38]. However, some reports showed that the proportion of these mutations depends on the location of GIST [7, 39]. In particular, among the exon 18 mutations, the *D842V* substitution is limited to GIST of the stomach [2, 36–38]. Also, in EGIST, the degree of *KIT* and *PDGFRA* mutations varies on where the location of the tumor is [3]. Namely, it is suggested that omental EGIST is similar to gastric GIST, mesenteric is similar to intestinal, and retroperitoneal can take either gastric or intestinal characteristics [11]. Within 57 omental EGIST cases we reviewed this time, 25 cases described about gene mutation; gene mutations of these cases were summarized and compared with GIST of other organs in Table 2 [7, 10, 14, 17, 19, 21, 24–26, 28, 39]. Mutation status in omental EGIST was very similar to gastric GIST. In particular, *PDGFRA D842V* mutation rate of omental EGIST seemed comparable with gastric GIST. These results suggested that omental EGIST is strongly related to gastric GIST, so the behavior of omental EGIST might be akin to gastric GIST.

**Table 2:** Mutation subtypes according to the primary location of GIST [7, 10, 14, 17, 19, 21, 24–26, 28, 39]

*Genetic type*	*Whole GIST* (%)	*Gastric GIST* (%)	*Intestinal GIST* (%)	*Omental GIST* (*our review*)
***KIT mutation***	**77**	**65.2**	**79.7**	**60% (15/25)**
*Exon 9*	8	1.8	23.0	4% (1/25)
*Exon 11*	67	61.4	54.0	56% (14/25)
*Exon 13*	1	1.2	2.3	―
*Exon 17*	1	0.8	0.4	―
***PDGFRA mutation***	**10**	**22.9**	**1.2**	**28% (7/25)**
*Exon 12*	1	3.1	0	8% (2/25)
*Exon 14*	< 1	0.5	0.4	―
*Exon 18 (D842V)*	5	19.3	0.8	20% (5/25)
***Wild type***	**13**	**11.9**	**19.1**	**20% (5/25)**

These results may provide some suggestions into the question of where omental EGIST originates. There are some hypotheses about the origin of EGIST: (1) they are derived from extraintestinal undifferentiated mesenchymal cells capable of differentiating to ICCs [14], (2) they originate from mesenchymal undifferentiated stem cells [13], and (3) they lose the extramural contact with the intestinal wall in GISTs [40]. However, their origin remains unclear due to their rarity. Further accumulation of EGIST cases and related studies is needed.

The standard treatment for GIST without metastasis is surgical complete resection. As for EGIST also, complete resection must be the only way to cure at present. As discussed above, omental EGIST has a similar clinicopathological and genetic background to gastric GIST. However, the prognosis of EGIST, including omental EGIST, after resection is thought to be less favorable than that of GIST [3, 9, 11]. -The reason is suggested that EGIST is often found in a very large state at the onset [9–11]. In fact, as shown in Table 1, the median tumor diameter at the onset of omental EGIST was 15 cm, extremely large. Because EGIST is not exposed to the GI tract, it is unlikely to show symptoms such as bleeding into the digestive tract or stenosis, and then the tumor detection tends to be delayed. And the larger the tumor grows, the worse some parameters predicting the prognosis, such as mitotic activity, cellularity, nuclear atypia, and necrosis, become, so it is convincing that the prognosis of EGIST, which is often found in larger states, is worse than that of GIST [27, 35].

The risk factors for recurrence of GIST are tumor size in the largest dimension > 5 cm and mitotic count > 5 per 50 HPF [2]. For GIST belonging to these high-risk groups, postoperative adjuvant imatinib therapy is known to improve prognosis [41, 42]. However, the response of GISTs to imatinib treatment depends on the mutation status. While most GISTs are sensitive to imatinib, those with *PDGFRA D842V* substitutions (exon 18) are resistant to imatinib [43, 44]. There is a report that the recurrence-free survival after curative resection in patients with *PDGFRA* mutations was more favorable than in patients with *KIT* mutations [37]. Owing to imatinib resistance and favorable prognosis, patients with GIST bearing *PDGFRA D842V* substitutions are not generally considered to be candidates for adjuvant imatinib therapy [44, 45]. As for EGIST, prognostic data is scarce and reported mutations are even more limited; hence, there is no consensus regarding the recommendations for adjuvant imatinib for this tumor. Prognosis of 5 cases who bare *PDGFRA D842V* substitutions in our review was 4–120 months (median 38 months) without recurrence and mortality. The prognosis seemed to be not so bad; however, the number of the cases was too small. Further studies are required to identify the risk factors of EGIST recurrence. At present, it is at best to deduce the results of GIST and apply them to EGIST.

In this case, we observed a mitotic count of < 1 per 50 HPF and Ki-67 labeling index of 3.3%. The estimated risk of tumor recurrence from these factors seemed to be relatively low [2, 46]. However, we emphasized the fact that the tumor was relatively large in 70 mm diameter (> 50 mm) and the tumor was EGIST (located outside the GI tract); we regarded this case as high-risk. As mentioned above, omental EGIST has a certain number of *PDGFRA* mutations; we previously performed a genomic examination to determine the indication of adjuvant therapy. Then, *PDGFRA D842V* substitutions were shown; this tumor was expected to be resistant to imatinib. Thus, the patient was not considered to be a candidate for adjuvant imatinib and was instead followed up frequently. Some TK inhibitors, such as avapritinib and crenolanib, had shown certain potent activities for GIST with *PDGFRA* mutations [47]. Early development of these drugs is expected.

## Conclusion

We described an extremely rare case of EGIST arising in the lesser omentum with a *PDGFRA* mutation. Our review showed that there were many clinicopathological and genetic similarities between omental EGIST and gastric GIST. However, the prognosis of omental EGIST was rather poorer than that of gastric GIST; much about EGIST, such as its origin and the effects of imatinib, still remains unknown. Further research is required.

## Data Availability

All data generated during this study are included in this published article.

## Abbreviations

GIST: Gastrointestinal stromal tumor
GI: Gastrointestinal
PDGFRA: Platelet-derived growth factor receptor alpha
TK: Tyrosine kinase
EGIST: Extra-gastrointestinal stromal tumor
EGD: Esophagogastroduodenoscopy
EUS: Endoscopic ultrasonography
CT: Computed tomography
HE: Hematoxylin-eosin
HPF: High-power fields
DOG-1: Discovered on GIST-1
ICC: Interstitial cells of Cajal
